# Editorial: Liquid biopsy—A great hope or just hype?

**DOI:** 10.3389/pore.2023.1611170

**Published:** 2023-03-28

**Authors:** Aleš Ryška

**Affiliations:** The Fingerland Department of Pathology, Charles University Medical Faculty, Hradec Králové, Czechia

**Keywords:** liquid biopsy, ctDNA, circulating tumor cells, exosomes, monitoring

## Introduction

Liquid biopsy is a term which gained in last decade a lot of attention from both clinicians (namely oncologists) and pathologists/scientists. It became one of the fancy buzzwords, creating a lot of expectations, sometimes without sufficient knowledge about its full potential, but more importantly (and certainly more frequently) about its limitations. Already the term liquid biopsy is quite misleading, as it covers several entirely different ways of detection of various genomic alterations in humans. Two most important—and mostly clinically used—modes are detection of circulating tumors cells (CTCs) and circulating cell free tumoral DNA (ctDNA). While the detection of genomic alterations in CTCs sounds more based on the tumor biology (we are detecting the changes in DNA in the cells, which are certainly neoplastic, are potential source of metastatic dissemination of the tumor and have survived at least the initial interaction with antitumor immunity), it turned out that this approach has so far only limited clinical application. The main reason are the technical/methodological challenges during the isolation of the CTCs as well as their limited number used for genomic testing. Considering the fact, that vast majority of them do not belong to the subgroup of tumor progenitor cells, which are the crucial players in tumor growth, we can easily detect the noise (mutations in cells which can represent merely a bystander information) dominating over the biologically/clinically important signal (genomic profile in tumor progenitor cells).

Detection of ctDNA, on the other hand, has been demonstrated to be sufficiently robust to be incorporated into everyday routine practice. The total amount of ctDNA statistically correlates with total tumor burden, represents genotype of neoplastic cells from multiple metastatic foci (and provides therefore more complex information about the tumor genotype), levels of ctDNA generally correlate well with response of tumor population to treatment and provide us with prognostic information. However, as devil is hiding in the detail, the two very important words in the lines above are “statistically” and “generally.” Multiple studies have demonstrated that ctDNA testing has several critical limitations, which are partially solvable and partially intrinsic. The solvable ones are the limited sensitivity of currently used methods and their costs. We see how much the technical and scientific progress improves the molecular genetic methods while the financial cost is dramatically decreasing every year. Thus, it is just question of time (at least this is what the author of this editorial believes) when the methods will reach such laboratory sensitivity and sufficiently low price which will be not anymore limiting their use in everyday practice. The other issue, however, are the inherent limitations, which cannot be solved by mere technological development. It is the well-known and repeatedly demonstrated fact that shedding of the ctDNA is not the same by all cancer types, differs (sometimes by order of several magnitudes) even in one diagnosis from patient to patient. Thus, the sensitivity of liquid biopsy is in most situations significantly lower than testing of the tumor tissue, irrespective of the fact, how sensitive method is used. On the other hand, with increasing the sensitivity of the method, we must face the other side of the coin (for long not the expected one)—the false positivity—which is the result of identification of mutations in cfDNA originating from elements of clonal hematopoesis.

The above statements should not demonstrate the skepticism of the author of this text, but rather the need for critical thinking and awareness of both pros but also cons of liquid biopsy.

Nevertheless, already today, liquid biopsy has found a way to routine clinical use in several settings and its potential is further massively investigated. To demonstrate how much has this term set in everyday medical practice, I tried to test (for the first time in my life) the publicly available system of artificial intelligence—ChatGPT (https://openai.com/blog/chatgpt/).

My question was very simple: What are the main pros and cons of liquid biopsy in testing genomic alterations of different types of cancer?

Here is the (rather complex and comprehensive) answer:

Liquid biopsy is a technique that involves the analysis of circulating tumor cells (CTCs), cell-free DNA (cfDNA), or exosomes, which are shed by tumors into the bloodstream. Liquid biopsy is a non-invasive method that allows for the detection of genomic alterations in cancer cells, which can provide important diagnostic and prognostic information, as well as help guide treatment decisions. Here are some of the main pros and cons of liquid biopsy in testing genomic alterations of different types of cancer.

### Pros

Non-invasive: Liquid biopsy is a non-invasive method that can be performed on a simple blood sample, which eliminates the need for invasive tissue biopsies.

Detection of genetic heterogeneity: Liquid biopsy can detect multiple genomic alterations in different regions of a tumor, which can help identify genetic heterogeneity within the tumor and guide treatment decisions.

Early detection: Liquid biopsy can detect cancer at an earlier stage than traditional imaging methods, which can lead to earlier treatment and better outcomes.

Monitoring disease progression: Liquid biopsy can be used to monitor disease progression and treatment response over time, which can help guide treatment decisions and identify resistance mechanisms.

### Cons

Sensitivity and specificity: Liquid biopsy may not always detect all genomic alterations present in the tumor due to limitations in sensitivity and specificity, which can result in false negatives or false positives.

Variability: The amount and quality of circulating tumor DNA can vary depending on the type and stage of cancer, as well as other factors, which can affect the accuracy of liquid biopsy results.

Cost: Liquid biopsy can be expensive, and may not be covered by all insurance plans.

Limited availability: Liquid biopsy testing may not be widely available, which can limit access to this technology for some patients.

In summary, liquid biopsy is a promising technique for the detection of genomic alterations in cancer, but it has limitations that need to be considered when interpreting the results. Further research is needed to optimize the sensitivity and specificity of liquid biopsy, as well as to establish its clinical utility in different types of cancer.

Not bad, right?

In this special issue of Pathology, Oncology and Research you find 8 articles focused on various aspects of liquid biopsy—from paper reporting routine implementation of LB in monitoring of the disease, as described by Benesova et al., through comparison of different commercially available detection kits in paper from Bencze et al. up to the experience with methodological challenges in detection of histone H3 K27M in cerebrospinal fluid as reported by Zaytseva et al. The article from Boniface and Spellman summarizes various factors (namely effect of treatment) responsible for variability of dynamics in ctDNA blood levels. Other papers are more oriented on future development—such as paper by Xia et al. describing single-cell transcriptome profiling signatures and alterations of microglia associated with glioblastoma multiforme or two papers focused on exploitation of exosomes as carriers of different subtypes of RNA (by Zhao et al. and by Zheng et al.). Finally, you will find very interesting data on potential use of detection of microRNA-181a combined with measurement of VEGF-A as an indicator as CNS involvement in children with leukemia. Enjoy reading this special issue!

